# A unique memory process modulated by emotion underpins successful odor recognition and episodic retrieval in humans

**DOI:** 10.3389/fnbeh.2014.00203

**Published:** 2014-06-06

**Authors:** Anne-Lise Saive, Jean-Pierre Royet, Nadine Ravel, Marc Thévenet, Samuel Garcia, Jane Plailly

**Affiliations:** Lyon Neuroscience Research Center, CNRS UMR 5292 - INSERM U1028 - University Lyon1Lyon, France

**Keywords:** episodic memory, recognition memory, encoding, olfaction, visuospatial context, emotion, breathing, human

## Abstract

We behaviorally explore the link between olfaction, emotion and memory by testing the hypothesis that the emotion carried by odors facilitates the memory of specific unique events. To investigate this idea, we used a novel behavioral approach inspired by a paradigm developed by our team to study episodic memory in a controlled and as ecological as possible way in humans. The participants freely explored three unique and rich laboratory episodes; each episode consisted of three unfamiliar odors (What) positioned at three specific locations (Where) within a visual context (Which context). During the retrieval test, which occurred 24–72 h after the encoding, odors were used to trigger the retrieval of the complex episodes. The participants were proficient in recognizing the target odors among distractors and retrieving the visuospatial context in which they were encountered. The episodic nature of the task generated high and stable memory performances, which were accompanied by faster responses and slower and deeper breathing. Successful odor recognition and episodic memory were not related to differences in odor investigation at encoding. However, memory performances were influenced by the emotional content of the odors, regardless of odor valence, with both pleasant and unpleasant odors generating higher recognition and episodic retrieval than neutral odors. Finally, the present study also suggested that when the binding between the odors and the spatio-contextual features of the episode was successful, the odor recognition and the episodic retrieval collapsed into a unique memory process that began as soon as the participants smelled the odors.

## Introduction

Human episodic memory is the memory that permits the conscious re-experience of specific personal events from the past (Tulving, 1972, 1983) and is associated with a feeling of mental time travel (Tulving, 2001, 2002). Because the investigation of this ability in animals is controversial, content-based approaches have been developed that focus on the different types of information stored in memory: *What* happened, *Where* and *When* (Clayton and Dickinson, 1998; Griffiths and Clayton, 2001; Babb and Crystal, 2006; Crystal, 2009). Subsequently, based on human phenomenological experiences of event recall, Easton and Eacott (2008; Eacott and Easton, 2010) enriched this refined definition of episodic memory. They widened its third dimension, replacing the temporal dimension with the specific occasion or context in which the event occurred, thereby leading to a “*What, Where, Which occasion*, or *Which context*” definition. The authors considered episodic memory as a “snapshot” of an episode in which time can form a part of the context but is not the only contextual marker. Emotion, semantic knowledge, the visual scene, or auditory and olfactory environments can also define the context of the episode. For example, when you remember the last time you went to a restaurant, you can recall where and when it was, as well as the occasion for which you were there, with whom, what you ate, and if you had a good evening. Importantly, these approaches did not consider the memory in terms of autonoetic consciousness, and therefore, were referred to as episodic-like memory (Clayton and Dickinson, 1998; Clayton et al., 2003).

In humans, two approaches are usually used to study past event retrieval. In the *ecological* approach, experimenters test autobiographical memory by interrogating participants about real-life memories encoded in their past (Fink et al., 1996; Levine et al., 2004; Piolino et al., 2004; Nadel et al., 2007; Janata, 2009). This approach is quite ecological because it is close to real-life recall, but the veracity of the recalled events cannot be controlled for. In the *laboratory-based* approach, experimenters test the memorization of artificial episodes created in the laboratory using recognition tasks (Konishi et al., 2000; Daselaar et al., 2003; Donaldson et al., 2010; Royet et al., 2011; Herholz et al., 2012), thereby permitting control of the encoding conditions, the retention time and the veracity of the retrieval. However, the information to be remembered is often one-dimensional (e.g., *What*) and is therefore poor in comparison with a real-life episode. To limit the drawbacks of such methods, new *laboratory-ecological* approaches halfway between these two traditional methods have recently been devised to explore human episodic memory (Pause et al., 2010, 2013; Holland and Smulders, 2011; Milton et al., 2011; Saive et al., 2013). We proposed such an intermediate approach that was deeply inspired by tasks developed to study episodic-like memory in animals to determine the experimental conditions that best evaluate episodic memory while remaining ecologically valid (Saive et al., 2013). This approach allowed the controlled study of trial-unique free encoding, retention delay and the retrieval of rich and complex episodes composed of unnamable odors (What) located spatially (Where) within a visual context (Which context).

Phenomenologically, olfaction, memory and emotion are closely linked. Odors are particularly evocative reminders of past events. Among all sensorial stimuli, odors trigger more vivid and emotional memories (Hinton and Henley, 1993; Herz and Cupchik, 1995; Chu and Downes, 2002; Larsson et al., 2009). This phenomenon can be explained because the functions of olfaction, memory and emotion involve anatomically tight brain areas. The primary olfactory cortex includes the piriform-periamygdaloid cortex, which gives way gradually to the lateral entorhinal cortex. From these areas, the olfactory signal is respectively transmitted to the amygdala and to the CA1 of the hippocampus (Price, 1973; De Olmos et al., 1978; Shipley and Reyes, 1991) before being sent to the secondary olfactory cortices, the orbitofrontal and insular cortices. Therefore, from its birth in the olfactory epithelium, the olfactory signal is relayed through two or three neurons to the brain structures critical for emotion and memory (for review, Eichenbaum, 2000; Sergerie et al., 2008). Despite some consensus on odor pleasantness especially for very pleasant and very unpleasant odors (Moncrieff, 1966), the emotion generated by odors can greatly differ between individuals (Ferdenzi et al., 2013). The differences in emotional responses to odors can result from variations in genetic backgrounds (Keller et al., 2007) but likely mainly result from differences in personal experience (Engen, 1991; Robin et al., 1998; Herz, 2001; Herz et al., 2004). The association between an odor and the emotional content in which it occurs determines its future hedonic tone and explains why the same odor can be perceived as either pleasant or unpleasant.

The objective of the current study was first to investigate the cognitive processes of episodic memory by combining in an original way the laboratory and autobiographical approaches. Second, it was to test the still-unexplored hypothesis that the emotion carried by odors facilitates the memory of specific unique events. To investigate this idea, we adapted our episodic memory task and addressed the episodic retrieval of episodes comprising three different odors positioned at specific locations within a visual context to create rich multidimensional episodes (Saive et al., 2013). To identify the differential influence of emotion on episodic memory, we tested the effects of emotion carried by odors on the behavioral and physiological responses of the participants during encoding and retrieval.

## Materials and methods

### Participants

Twenty-five healthy participants [13 women; age: 21.4 ± 2.1 years (mean ± standard deviation)] consented to participate in the experiment. All participants were right-handed and reported normal senses of smell and no visual impairments. They provided written informed consent as required by the local Institutional Review Board in accordance with French regulations for biomedical experiments with healthy volunteers [Ethical Committee of CPP Sud-Est IV (CPP 11/007), ID RCB: 2010-A-01529-30, January 25, 2011] and received financial compensation. The study was conducted in accordance with the Declaration of Helsinki.

### Stimuli and materials

#### Odorants

Eighteen odorants consisting of essential oils and single or mixtures of monomolecular chemical compounds were selected *a priori* based on their distinctiveness and relatively low identifiability and familiarity. The odorants were subdivided into two sets (Sets 1 and 2) of nine odors each. Set 1 was composed of butanol, calone, carrot, cis-3-hexenyl salicylate, dihydromyrcenol, methyl octine carbonate, musk, rosemarel and stemone. Set 2 was composed of allyl amyl glycolate, basil, birch oil, citronellol, ethyl acetyl acetate, linalyl acetate, rose oxide, styrallyl acetate and tobacco.

The odorants were presented using a 20-channel computer-controlled olfactometer adapted from an olfactometer previously described by Sezille et al. (2013). Briefly, this odor diffusion system was developed to synchronize odorous stimuli with breathing. Undiluted odorants were contained in a 10-ml U-shaped Pyrex® tube (VS Technologies, France) filled with odorized microporous substances. Odorized airflows and air carrier were sent to and mixed in a homemade mixing head made of polytetrafluoroethylene and connected to the nostrils. The participant's respiratory signal was acquired using a nasal cannula and was used to trigger the odor stimulation through an airflow sensor. The airflow rate was set at 3 l/min, and the odorants were delivered over 4 s.

#### Spatio-contextual environment

**Figure 1:**
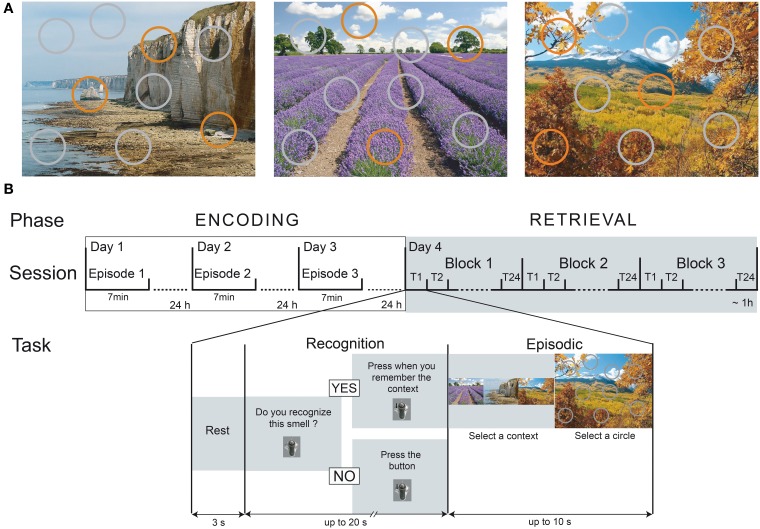
**Episodic-memory task design. (A)** The three spatio-contextual environments of the episodes. Orange circles represent the spatial locations associated with an odor. **(B)** The temporal course of the encoding and retrieval sessions. During the encoding, the participants discovered one episode per day over 3 days. On the fourth day, the memory of the episodes was tested using an odor-recognition task followed for the “Yes” trials by an episodic memory retrieval. T, Trial.

The spatio-contextual environment was presented within the experimental setup previously described by Saive et al. (2013), but modified for the present study. Three landscape pictures presented full-screen (1280 × 1024 pixels, 72 dpi) constituted the visual contexts (a coastal cliff, a lavender field and a mountain landscape; Figure 1A). For each of the three contexts, circles symbolized nine spatial locations: 6 were colored in gray, and 3 were colored in orange. When the circle was orange, it was associated with an odor; otherwise, it was gray. All spatial locations of the orange circles and all odors differed between the contexts.

#### Multidimensional episodes

Three multidimensional episodes were created, which were each composed of three odors (What) associated with specific locations (Where) within a given visual context (Which context). Three multidimensional episodes were created, which were each composed of three odors (What) associated with specific locations (Where) within a given visual context (Which context). To limit associative semantic processes, the odors, spatial locations and visual context were arbitrary linked.

**Figure 2:**
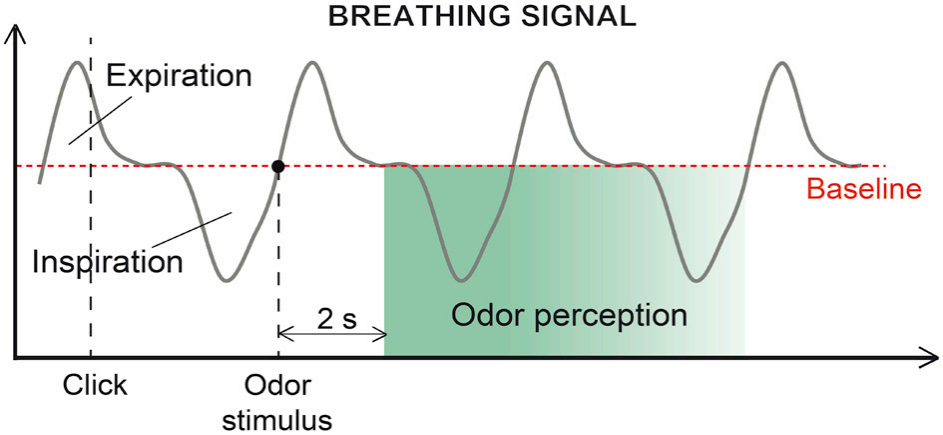
**Breathing signal**. Course of a typical breathing signal depicting successive expirations and inspirations. The odor was sent at the beginning of the participant's expiration to ensure odor perception at the beginning of the next inspiration, which occurred approximately 2 s later (in green, period of odor perception, fading with time).

An in-house LabView software (version 8.6 or higher) controlled the presentation of odors, pictures and circles and recorded the participants' responses and breathing throughout the experiment. The participants were requested to breathe normally and avoid sniffing behaviors (Figure 2). To interact with the software, the participants used a trackball (Kensington, Redwood Shores, CA, USA). When the participants clicked on a circle, the odor stimulus was delivered at the beginning of the subsequent expiration, enabling the odor to be perceived at the beginning of the next inspiration (on average 2 s later). The volume, amplitude and duration of each inspiratory cycle were recorded, and the respiratory frequency was calculated.

### Experimental procedure

The experimental procedure consisted of four sessions performed over the course of 4 successive days. The first three sessions were used for encoding, and the retrieval occurred in the fourth session (Figure 1B). A full night of sleep followed each of the encoding sessions to promote consolidation and to reduce interference (Maquet, 2001; Stickgold, 2005). Participants completed the four sessions at the same time of the day to limit the differential influence of internal states (hunger, satiety) on olfactory and cognitive processes between sessions (Jiang et al., 2008; Plailly et al., 2011).

There were two groups of participants: G1 and G2. For G1, the Set1 odorants were defined as the targets, and the Set2 odorants were defined as the distractors. For G2, the Set2 odorants were defined as the targets, and the Set1 odorants were defined as the distractors.

#### Encoding

During encoding, the participants freely discovered one episode per day for 7 min (Figure 1B). They were asked to explore all dimensions of the episode as much as possible by paying attention to the background picture, the circles superimposed on this background, and the odors that are delivered when clicking on the orange circles. No memorization instruction was given, thereby ensuring free encoding, similar to what arises in real-life situations. The participants were only informed that they would be questioned about their perception of the episodes on the fourth day. The order of the three episodes was randomized between the participants.

#### Retrieval

Retrieval was performed on the fourth day. The session consisted of three blocks of 24 trials, and each block corresponded to the presentation of 15 target odors and 9 distractor odors. Each target odor was presented five times, and each distractor odor was presented three times. For a given block, the target and distractor odors were presented in a pseudorandom order such that two presentations of the same odor were separated by at least two trials. The odor presentation order was counterbalanced between the participants.

Each trial began with an odor recognition task (Figure 1B). The participants were presented the odors and had to determine whether they recognized the smell (“*Do you recognize this smell?*”) as having been previously presented during the encoding. Two situations could happen. 1) If the participants responded “*Yes*,” they were then asked to retrieve the entire episode associated with the odorant and to press on the trackball if they succeeded in less than 20 s after the odor was sent (“*Press when you remember the context*”). After this delay, they were given up to 10 s to choose both the accurate visual context and the exact location of the odor by selecting one of the three pictures, followed by one of the nine circles. A response was considered correct when the participants selected both the accurate context and the specific location previously associated with the odor during the encoding. 2) If the participants responded “*No*,” they had to press on the trackball (“*Press the button*”) and rest until the next trial.

Following this retrieval task, the strength of the association between the spatial location and the visual context of an event was tested. The participants had to recall the three locations (orange circles) associated with the odors in every visual context during the encoding.

#### Rating of odor intensity, pleasantness, and familiarity

At the end of the experiment, the participants were asked to rate the odorants in terms of intensity, pleasantness and familiarity using non-graduated scales. The pleasantness scale was divided into two equal parts by a “*neutral*” value separating the ratings of unpleasantness and pleasantness. The intensity, pleasantness and familiarity ratings were *a posteriori* transformed into scores from 0 to 10.

### Data analysis

#### Encoding

For each participant, the number of clicks was computed per odor. For each odor, the time periods between two consecutive clicks (delay) were measured, and the mean delay was then determined. The time window between the two clicks served as the time frame for the analyses of breathing parameters (e.g., the volume, amplitude and duration of the inspiratory cycles and the respiratory frequency). The influence of the odor characteristics (intensity, pleasantness and familiarity) on the behavioral and physiological (breathing) data was tested. The relationship between the encoding and the retrieval was investigated by analyzing the behavioral and physiological data during the encoding as a function of the subsequent memory performances.

#### Retrieval

Recognition memory performance was assessed using parameters from the signal detection theory (Lockhart and Murdock, 1970). From the experimental conditions (target vs. distractor) and the participants' behavioral responses (“*Yes*” vs. “*No*”), four response categories were defined: Hit and Miss occurred when the target items were accurately recognized or incorrectly rejected, respectively, and correct rejection (CR) and false alarm (FA) occurred when the distractor items were correctly rejected or incorrectly recognized, respectively. In the framework of the signal-detection theory, a memory score (*d*'_*L*_) reflected the participant's ability to discriminate between the target and distractor items. This score was determined from the Hit and FA scores and was calculated as follows:

$${d^{\prime }}_{L}=\ln\frac{HR(1-FR)}{FR(1-HR)}$$

Where *HR* represents the Hit rate [(Hit + 0.5)/(*N_t_* + 1)], *FR* represents the false alarm rate [(FA + 0.5)/(*N_d_* + 1)] and *N_t_* and *N_d_* represent the number of target and distractor odors, respectively, for which the participants provided an answer. Memory scores may be good or poor (positive or negative values, respectively).

In the episodic retrieval test, we focused the analyses on the participants' accurate responses for the target odors (Hit). Four types of responses were then defined depending on the recall accuracy. When the participants correctly recognized the target odors, they could accurately remember both the location and the context (WWW), the location only (WWhere), or the context only (WWhich) or they could be mistaken about both dimensions (What). These different scenarios were named *episodic combinations*. The theoretical proportions of these episodic combinations resulting from responses given randomly were 0.019 for WWW [1 response (“*Yes/No*”) out of 2 * 1 context out of 3 * 1 location out of 9], 0.148 for WWhich [1 response (“*Yes/No*”) out of 2 * 1 context out of 3 * 8 locations out of 9], 0.037 for WWhere [1 response (“*Yes/No*”) out of 2 * 2 contexts out of 3 * 1 location out of 9] and 0.296 for What [1 response (“*Yes/No*”) out of 2 * 2 contexts out of 3 * 8 locations out of 9].

The response times for odor recognition and episodic retrieval were considered. The response times corresponded to the durations between the first inspiration after the odor was delivered and 1) the “*Yes/No*” response for the odor recognition task and 2) the “*I remember the context*” response for the episodic retrieval task. The same time boundaries were used to record and analyze breathing parameters during the odor recognition and episodic retrieval tasks.

### Statistical analysis

Behavioral and physiological data were z-scored [(x−μ)/σ] at the individual level to remove bias based on inter-individual differences. The number of each response given during the odor recognition and episodic retrieval tasks was further normalized by the number of trials after removal of one odor *a posteriori* from the data (“*Odor intensity, pleasantness and familiarity*”). The statistic main effects of the factors and interactions were determined using repeated measurements ANOVAs followed by *post-hoc* bilateral Student *t*-tests when main effects and/or interactions were significant. The effects were considered significant at *p* < 0.05. The relation between perceptual ratings of odors (intensity, pleasantness, familiarity) or memory performances with behavioral measures (number of clicks, delay between clicks) or breathing parameters was tested using Pearson tests. In these cases, to control for the Type I error rate associated to multiple comparisons, we applied the Bonferroni correction by dividing the probability alpha by the number of comparisons. Statistical analyses were performed using Statistica (StatSoft®, Tulsa, OK, USA).

## Results

### Odor intensity, pleasantness, and familiarity

On average, the odorants were perceived as moderately intense (5.31 ± 1.44; range: 1.49–7.15), relatively neutral (4.85 ± 1.38 range: 2.22–6.92) and unfamiliar (4.54 ± 1.61; range: 1.60–7.33). The intensity of the allyl amyl glycolate was rated as weak (1.49 ± 1.93) when compared with that of the other odorants. The Grubbs test, which was used to test for outliers, indicated that this intensity value abnormally deviated from the mean (*G* = 2.66, *p* = 0.04). As a consequence, the data related to allyl amyl glycolate were excluded from further analyses.

### Memory performances

The effects of the set of target odors (Set1 vs. Set2) selected for the participants of G1 and G2 and of the age of the episodes (1–3 days) on the behavioral and breathing responses observed during the encoding and retrieval sessions were evaluated. The influence of the repetition of the odors (5 times for targets and 3 times for distractors) on memory performances, response times, and breathing during retrieval was also tested. No significant main effects or interactions were found, and thus we did not take these factors into account in the subsequent analyses. Second, as the effect of context (coastal cliff, lavender field, and mountain landscape) was confounded with the nature of the three odors associated with each context, we could not specifically analyze it.

#### Encoding

The investigation of the odors during the encoding was analyzed as a function of the odor characteristics. The participants smelled, on average, each odor 5.5 (±2.6) times by clicking on the circles. The number of clicks for each odor for all participants was significantly negatively correlated with the odor intensity [*r* = −0.22, *t*_(1,210)_ = 3.30, *p* = 0.001, α_adjusted_ = 0.017] but not the odor familiarity and pleasantness (*p*_s_ > 0.11). The mean delay between the two odor investigations was 29.8 (±13.5) s. These delays were not correlated with the intensity, pleasantness, or familiarity of the odors (*p*_s_ > 0.05, α_adjusted_ = 0.017). The duration, amplitude and volume of the inspirations and the respiratory frequency did not vary significantly as a function of the odor's intensity, pleasantness and familiarity (*p*_s_ > 0.04, α_adjusted_ = 0.017).

#### Odor recognition

**Figure 3:**
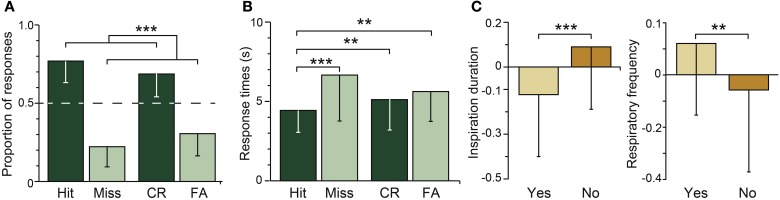
**Odor recognition. (A)** Mean distribution and **(B)** mean response times of correct (black) and incorrect (gray) responses for the target (Hit, Miss) and distractor (CR, FA) odors. **(C)** Mean normalized inspiration duration and respiratory frequency during odor recognition (“*Yes*” responses) and odor rejection (“*No*” responses). The dashed horizontal line indicates the random level. Vertical bars represent the SD. ^**^ *p* < 0.01; ^***^ *p* < 0.001.

The participants were presented the target and distractor odors and were asked whether they had smelled them during the encoding phase. The memory score was high (*d*'_*L*_ = 2.33 ± 1.18), which indicated that the participants were very proficient in recognizing old odors and rejecting new ones. The proportions of the different response categories (Hit, Miss, CR, and FA) are shown in Figure 3A. The proportion of correct responses (Hit + CR) was significantly higher than the proportion of incorrect responses (Miss + FA) [*F*_(1, 24)_ = 135.29, *p* = 0.0001]. While odor type (target vs. distractor) and response accuracy significantly interacted [*F*_(1, 24)_ = 4.11, *p* = 0.045], no significant differences were observed between Hit and CR and between Miss and FA (*p*_*s*_ > 0.06).

Figure 3B represents the influence of response accuracy (correct vs. incorrect) and odor type (target vs. distractor) on the response times. Response accuracy [*F*_(1, 24)_ = 29.33, *p* = 0.001] but not odor type [*F*_(1, 24)_ = 1.98, *p* = 0.17] significantly impacted the response times; the participants responded more rapidly when answering accurately (Hit + CR: 4.75 ± 1.71 s) than inaccurately (Miss + FA: 6.10 ± 2.44 s). Response accuracy and odor type significantly interacted [*F*_(1, 24)_ = 9.17, *p* = 0.004]; the participants gave correct responses more rapidly than incorrect responses when the target odors were presented (*p* = 0.001) but not when the distractor odors were presented (*p* = 0.19). The participants also answered more rapidly for the Hit responses than for the Miss, CR, and FA responses (*p_s_* < 0.001).

The breathing variations were analyzed as a function of response accuracy and odor type. No significant effects of response accuracy and odor type on the duration, amplitude and volume of the inspiration (*p*_*s*_ > 0.23) or the respiratory frequency (*p* = 0.07) were found. However, a significant interaction was identified between both factors and the duration [*F*_(1, 24)_ = 13.85, *p* = 0.001] and respiratory frequency [*F*_(1, 24)_ = 7.51, *p* = 0.008] but not the amplitude and volume of the inspirations (*p*_*s*_ > 0.18). As shown in Figure 3C, the duration of the participants' breath was shorter and their respiratory frequency was higher when they recognized the odors (“*Yes*” responses: Hit, FA) than when they rejected them (“*No*” responses: Miss, CR).

The recognition performances did not depend on the exploratory behavior of the odors during the encoding. The number of accurate odor recognitions (Hit) was not correlated with the number of clicks (*p* = 0.62, α_adjusted_ = 0.025) and the mean delay between the clicks (*p* = 0.62, α_adjusted_ = 0.025).

#### Episodic retrieval

**Figure 4:**
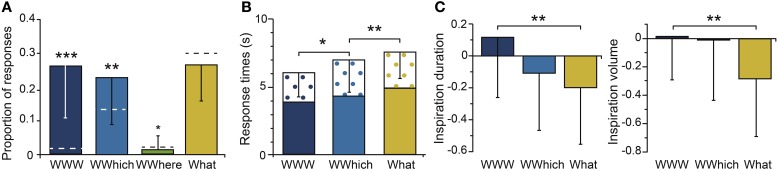
**Episodic retrieval. (A)** Mean proportions of episodic combinations (WWW, WWhich, WWhere, What). **(B)** Mean response times for each episodic combination, with the delay between the odor recognition and episodic retrieval responses represented in black crosses. **(C)** Mean normalized inspiration duration and volume for each episodic combination. The dashed horizontal lines indicate the random levels computed for the episodic combinations. Vertical bars represent the SD; ^*^ *p* < 0.05; ^**^ *p* < 0.01; ^***^ *p* < 0.001.

When the participants recognized an odor as the target, they were asked to retrieve the spatio-contextual environment in which it occurred. We focused our analysis on the responses following correct odor recognition (Hit). The proportions of the episodic combinations are represented in Figure 4A. The proportions of WWW, WWhich and What were significantly higher than the proportion of WWhere [*F*_(3, 66)_ = 20.55, *p* = 0.001; *post-hoc*, *p_*s*_* < 0.001]. The proportions of complete accurate (WWW) and partially accurate responses (WWhich, WWhere) that were given by the participants differed significantly from the random responses (*p_*s*_* < 0.017), while the proportion of inaccurate responses (What) did not differ from the proportion of random responses (*p* = 0.19). Thus, the participants were able to retrieve the spatio-contextual environment of the episodes using the recognition of an odor, they recalled only a part of the episode, or they did not recall anything and responded randomly. The subsequent analysis did not include the responses associated with the WWhere episodic combination because of the small amount of data.

The response times were then analyzed (Figure 4B). A significant main effect of the episodic combinations was found [*F*_(2, 46)_ = 18.56, *p* = 0.001]. The response times of the participants were significantly faster for perfect accurate responses (WWW) than for partially inaccurate responses (WWhich: *p* = 0.016). The response times were even faster for WWhich than for inaccurate What responses (*p* = 0.001). In other words, the more incorrect the answers, the slower the participants answered. Interestingly, the time interval between the odor recognition and the episodic retrieval responses did not significantly vary with the episodic combinations [*F*_(2, 46)_ = 2.11, *p* = 0.14].

The mean durations and volumes of the inspirations are given for the episodic combinations WWW, WWhich and What in Figure 4C. These durations and volumes significantly varied with the episodic combinations [*F*_(2, 46)_ = 5.31, *p* = 0.008 and *F*_(2, 46)_ = 4.88, *p* = 0.011, respectively]. The duration and volume of the inspirations were greater when the participants remembered the spatio-contextual environment associated with the odor (WWW) than when they did not remember it (What, *p*_*s*_ < 0.001). No significant differences in the respiratory frequency and amplitude of the inspirations were observed (*p*_*s*_ > 0.15).

The influence of the exploratory behavior of odors during encoding on the episodic performances was investigated. The number of accurate episodic retrievals (WWW) was not correlated with the number of clicks (*p* = 0.70), and the mean delay between clicks (*p* = 0.69).

Following this episodic retrieval, the strength of the association between the spatial location and the visual context of an episode was tested. On average, the participants accurately recollected 80 ± 7% of the spatial locations associated with each visual context. These performances did not significantly depend on the visual context [*F*_(2, 46)_ = 1.76, *p* = 0.19], which indicated that no difference in the strength of the visuospatial associations biased the episodic performances.

### Influence of emotion

**Figure 5:**
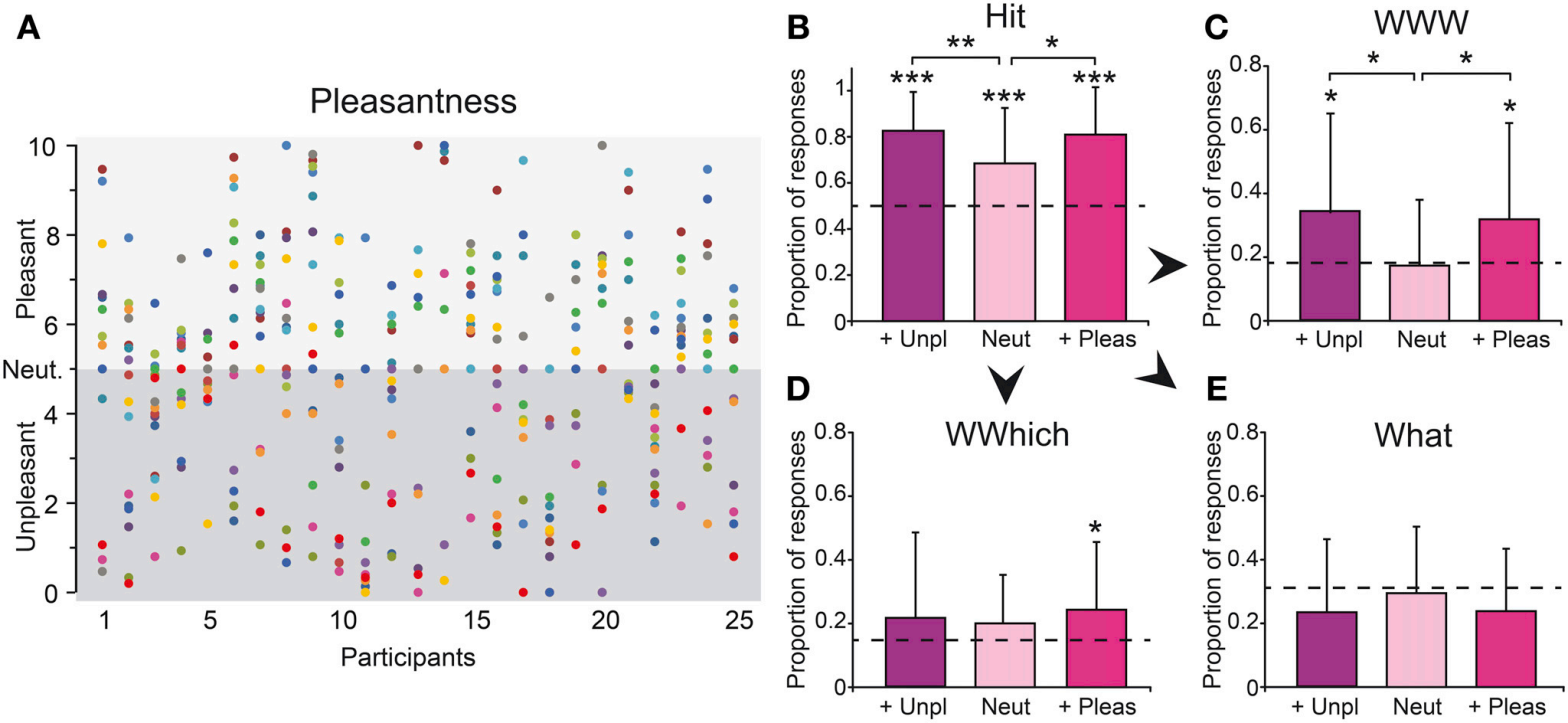
**Emotion. (A)** Pleasantness ratings of the 17 odors for the 25 participants. Each odorant is represented by a different color. Number of **(B)** Hits, **(C)** WWWs, **(D)** WWhichs, and **(E)** Whats as a function of the odor's pleasantness (more unpleasant, neutral and more pleasant). Unp, unpleasant; Neut, neutral; Pleas, pleasant. The dashed horizontal lines indicate the random levels computed for each response. Vertical bars represent the SD; ^*^ *p* < 0.05; ^**^ *p* < 0.01.

To investigate the influence of emotion on the memory performances, we created three odor pleasantness categories. Given that the pleasantness ratings of the odors widely varied among the participants (Figure 5A), we selected the two more pleasant, the two more neutral and the two more unpleasant odors for each participant. The odors selected for these three pleasantness categories differed significantly in terms of intensity [*F*_(2, 46)_ = 15.14, *p* = 0.001] and familiarity [*F*_(2, 46)_ = 20.37, *p* = 0.001]: the unpleasant odors were perceived as more intense and less familiar (6.36 ± 1.85; 3.05 ± 2.20, respectively) than the neutral odors (4.25 ± 2.01; 3.74 ± 2.38, respectively), while the pleasant odors (6.29 ± 1.45; 6.69 ± 2.09, respectively) were perceived as more intense and familiar than the neutral odors (*p_s_* < 0.001).

#### On memory performances

During the encoding, the number of clicks and the mean delay between two clicks did not differ between the pleasantness categories (*p*_*s*_ > 0.71), indicating that the emotions carried by the odors did not influence their exploration.

The proportions of correct recognition (Hit) of odors differed significantly from the random responses whatever the emotion of odors (*p*_*s*_ < 0.002), but it significantly varied as a function of the pleasantness category [*F*_(2, 46)_ = 5.42, *p* = 0.007; Figure 5B]. The pleasant and unpleasant odors were recognized more accurately than the neutral odors (*p* = 0.024 and *p* = 0.003, respectively).

Considering episodic retrieval performances, the proportions of complete accurate responses (WWW) differed significantly from the random responses when triggered by pleasant and unpleasant (*p*_*s*_ < 0.042) but not neutral odors (*p* = 0.72). The proportion of partial accurate responses (WWhich) significantly varied from random responses when triggered by pleasant odors only (*p* = 0.042; neutral and unpleasant odors, *p*_*s*_ > 0.12), while the proportion of inaccurate responses (What) did not differ from the proportion of random responses whatever the pleasantness category of the odors (*p*_*s*_ > 0.20). We observed a significant effect of the pleasantness category on the number of accurate episodic retrieval (WWW) responses [*F*_(2, 46)_ = 3.27, *p* = 0.046, Figure 5C] but not on the number of partial episodic retrieval (WWhich, Figure 5D) or inaccurate episodic retrieval (What, Figure 5E) responses (*p_s_* > 0.56). The number of WWW was significantly higher when the odors that triggered the memory were more pleasant or more unpleasant than neutral (*p* = 0.047 and *p* = 0.024, respectively). No significant difference was found between the pleasant and unpleasant odors (*p* = 0.79). Thus, the emotion carried by the odors only improved the retrieval of accurate episodic memories, regardless of the positive or negative valence of the emotion. Importantly, while odor pleasantness categories differed in terms of familiarity and intensity, the accurate odor recognition (Hit) and episodic retrieval (WWW) performances were not significantly related to these ratings (*p*_*s*_ > 0.49).

#### On response time and breathing

Regardless of the performances, the participants answered with similar response times regardless of the pleasantness category of the odors during odor recognition [*F*_(2, 46)_ = 0.97, *p* = 0.39] and episodic retrieval [*F*_(2, 46)_ = 1.26, *p* = 0.30]. Regardless of the performances, the participants answered with similar response times regardless of the odor pleasantness category during odor recognition [*F*_(2, 46)_ = 0.97, *p* = 0.39] and episodic retrieval [*F*_(2, 46)_ = 1.26, *p* = 0.30]. Performing two-way Session x Category ANOVAs on breathing data, we found a significant effect of pleasantness category on inspiration volume and duration [*F*_(2, 48)_ = 5.42, *p* = 0.008 and *F*_(2, 48)_ = 5.66, *p* = 0.006, respectively], and significant effects of pleasantness category and sessions on respiratory frequency [*F*_(2, 48)_ = 3.34, *p* = 0.044 and *F*_(2, 48)_ = 6.56, *p* = 0.003, respectively]. No significant effect was found for amplitude, and no significant interaction between factors was found whatever the breathing parameters. Thus, participants inspired more deeply, with longer inspirations, and less frequently for neutral and pleasant odors than unpleasant odors, whatever the session (*p*_*s*_ = 0.017). They inspired also less frequently during episodic retrieval than during encoding (*p* = 0.018).

## Discussion

The present novel laboratory-based episodic memory approach, which was adapted from a previous paradigm developed by our team (Saive et al., 2013), succeeded in the formation and subsequent retrieval of an integrated and multimodal memory of episodes comprising odors (What) localized spatially (Where) within a visual context (Which context). Successful odor recognition and episodic memory were not related to differences in the odor investigation at encoding and were paralleled by modifications in both the response time and breathing patterns. However, memory performances were influenced by the emotional content of the odor, with both pleasant and unpleasant odors generating higher recognition and episodic retrieval than neutral odors.

### Recognition and episodic memory processes

The behavioral data revealed a high ability to recognize odors previously encountered in laboratory settings. The unfamiliar odors freely encoded during episode discovery were proficiently recognized among the new odors encountered afterwards, as indicated by a very high memory score. The good memory recognition performances were supported by the behavioral measures. The participants answered more rapidly when they successfully recognized the target odors than for all the other responses. Moreover, the duration of the participants' breath was shorter and their respiratory frequency was higher when they accurately recognized the odors than when they rejected them. These response times and breathing observations are consistent with previous reports (Jehl et al., 1997; Olsson and Cain, 2003; Royet et al., 2011) and could be evidence for a serial identity matching process between the memory trace and the actual percept (Bamber, 1969). Until a match was found between the odor cue and the odor memory traces, the participants needed to follow the memory search (which ended in higher response times for *No* than *Yes* responses) and keep the odor “in their nose,” which led to expanded respiratory cycles. These results demonstrate the efficiency of our paradigm in generating the encoding of unknown odors and their later recognition.

The old odors were not only very well recognized but they also triggered the retrieval of past unique episodes at a level far above chance. From the accurate recognition of an odor, the participants were able either to retrieve the complete visuospatial context of the episodes or correctly recall only the context of the episodes. Otherwise, they did not remember any information related to the episode and answered randomly. Two scenarios are possible to explain the cognitive processes engaged in episodic retrieval: a serial recollection of the three dimensions (What, Where, and Which context) or an immediate recall of the whole episode. In the first scenario, when an odor was recognized, the participants interrogated their memories until the exact position of the odor in the exact context was recalled. In the second scenario, the episode was fully recovered from odor perception, all of its dimensions at once. The analysis of the response times revealed that the more information the participants retrieved about the episode, the faster they answered. However, the time period between odor recognition and episodic retrieval remained constant regardless of the accuracy of the episodic retrieval; this finding suggests that the content of the memory was already fully recovered from the odor recognition or that the episodic retrieval was already fairly advanced. Therefore, the response time data more strongly support the retrieval of the whole episode at once rather than a serial recall of its dimensions. The detailed analyses of the cognitive processes involved in our paradigm led us to support for the collapse of the recognition and episodic retrieval processes into a unique memory retrieval process when the binding between the odors and the spatio-contextual features of the episode is successful. The odor perception might generate the simultaneous recognition of the odor and the recall of other episodic features, such as the characteristics of the odor, the localization of the orange circle on the visual background or the mood the participants were in. These memories seem to be triggered as soon as the participants smelled the odor. Therefore, the odor recognition of the odor would be included in the episodic retrieval as one feature of the episode. Otherwise, when unsuccessful, the recognition and episodic retrieval memory process might be distinct.

Recognition and episodic performances were independent of the way the odors were investigated at encoding and the odors' intrinsic characteristics. The only exception was the odors that were less intense and were investigated more often, most likely to better characterize them. Given the amount of evidence indicating a serial position effect on recognition memory, with first and more recent items more likely to be recognized (Deese and Kaufman, 1957; Murdock, 1962), as well as on autobiographical memory, with events from late childhood or young adulthood and recent events more likely to be remembered (Crovitz and Schiffman, 1974; Crovitz and Quina-Holland, 1976), we might have expected primacy and recency effects to be observed. However, our data demonstrated that odor recognition and episodic memories were similar whether the day of encoding was the first, second or the last day, thereby confirming previous results (Saive et al., 2013). Thus, these performances were stable over time and were not dependent on the age of the retrieved episode. Furthermore, the performances were not impacted by the multiple presentations of the odors during the retrieval phase, although it has been demonstrated that repeated presentations of odors increase their familiarity (e.g., Jehl et al., 1995). These high and stable memory performances might reflect the influence of the multimodality and the episodic nature of our task. Odors are better recognized when associated with indices of other modalities or when associated with an episode of life during encoding (Lyman and McDaniel, 1986, 1990). When exploring the episodes, the participants were experiencing a new, rich and complex event, very similar to real-life encoding situations, which enhanced the strength of the whole memory trace. The full nights of sleep obtained between the encoding sessions may also have strengthened the consolidation of the memory traces and limited the interference between the episodes (Maquet, 2001; Stickgold, 2005; Alger et al., 2012; Abel and Bäuml, 2014).

Odors that triggered the retrieval of the spatio-contextual environment were associated with increased duration and volume of inspirations compared with odors that did not trigger any recall. These data are consistent with previous studies investigating breathing during autobiographical retrieval (Masaoka et al., 2012a,b). The current variation in breathing during memory construction raises interesting questions. Were the physiological responses a consequence of a successful episodic search or were they necessary for the search to be successful? In other words, were the breathing characteristics modified by the retrieval of the elements of the episodes or did they reflect an intense memory search? These questions are reminiscent of findings that showed attention and mental imagery processes are associated with larger sniffs when participants succeed in the tasks (Bensafi et al., 2003, 2005; Plailly et al., 2008). It is further possible that the reconstruction of the memory necessitated a relaxed state that was reflected in slower respiration. A previous study showed that yoga breathing specifically increased spatial memory performances (Naveen et al., 1997).

### Impact of emotion generated by odor on memory retrieval

Compared to neutral odors, both pleasant and unpleasant odors generated increased recognition and more complete episodic retrieval. This suggests that the intensity of the emotion, also called emotional arousal, but not the valence (pleasant vs. unpleasant) enhanced memory retrieval. Many studies have indicated an emotional arousal benefit on memory in humans (Burke et al., 1992; Cahill and McGaugh, 1995; Laney et al., 2004). For example, Cahill and McGaugh (1995) have shown that the higher the arousal content of a story, the better the long-term retention. This beneficial aspect of human memory would be highly adaptive, enabling more efficient accessibility of emotional memory, and is strongly dependent on the amygdala (Hamann, 2001). Interestingly, the effect of emotion on accurate odor recognition was in fact only observed when the complete episode was accurately recalled. Incomplete or inaccurate recalls of the episodes were not influenced by emotion. The fact that the accurate recognition of the odor and the accurate retrieval of the episodes were affected the same way by emotion is another argument favoring the idea that, in the case of an efficient episodic retrieval, these two memory processes might be collapsed into a unique memory process.

When did emotion influence episodic memory? Emotion can modulate the creation, storage and recollection phases of episode processing (Holland and Kensinger, 2013). First, arousing items are noticed quickly, and attention is preferentially directed toward them, potentially promoting their encoding (Kensinger and Corkin, 2004; MacKay et al., 2004; Leclerc and Kensinger, 2008). Furthermore, both pleasant and unpleasant odors trigger the modulation of skin conductance and heart rate measures (Alaoui-Ismaïli et al., 1997a,b; Bensafi et al., 2002; Royet et al., 2003). Thus, in the present study, the odors might have generated automatic emotional responses that might have modulated the participant's attention and induced improved encoding of all associated information. Second, emotional arousal could also influence the memory consolidation. Indeed, it has been shown that sleep not only promotes the general consolidation of new acquired memory traces (Maquet, 2001; Stickgold, 2005) but also specifically supports emotional memories (Wagner et al., 2006; Holland and Lewis, 2007; Groch et al., 2013). Finally, emotion can modulate retrieval by increasing how easily the memory comes to mind following cue perception and by increasing the amount of remembered details (Kensinger, 2009; Melcher, 2010). In the current experiment, odor pleasantness influenced the accurate retrieval of olfactory episodes. Importantly, odor pleasantness did not differentially impact the exploratory behavior (number of clicks and delays between clicks) during encoding and its influence on breathing did not differ between sessions. Therefore, in the frame of the experimental conditions of our study, we can suggest that odor pleasantness had only an impact on the consolidation or memory retrieval but not on the encoding of the episodes.

Which memory process was influenced by emotion? In our case, the emotion triggered by odors enhanced both the odor recognition itself and the retrieval of the entire episode. Emotional arousal enhances the binding of contextual details or dimensions when they are an integral part of the emotional stimulus (Mather, 2007; Mather and Nesmith, 2008; Nashiro and Mather, 2011). In our study, we suggest that the dimensions of the episodes were encoded as features of the emotional odors and were combined in an integrated unique memory trace. Taken together, remembering how the features of an event were associated together is a critical aspect of episodic memory that seems to be promoted by emotion.

In conclusion, our study represents the first laboratory-ecological approach involving olfactory dimension that allows the conscious and controlled recollection of specific and complex events from the past. It combines in a very original way the advantages of the laboratory-based approaches that allow the control of encoding and recall conditions, and of autobiographical-based approaches that enable the retrieval of real life episodes (Saive et al., in revision). Furthermore, of interest to the entire neuroscientist community devoted to the study of memory, our paradigm enables the ecological and direct comparison between episodic and recognition memory processes, rather than indirect assessment based on the comparison between recollection and familiarity processes engaged in simpler memory tasks.

It demonstrates that humans are capable of encoding and remembering rich and unique laboratory episodes triggered by odors. The episodic nature of the task generates high and stable memory performances, accompanied by slower and deeper breathing. It shows for the first time that the emotion carried by odors, regardless of their valence, does not influence encoding behavior but promotes their accurate recognition and the accurate retrieval of the visuospatial context of the episodes. Importantly, this study also suggests that when the binding between the odors and the spatio-contextual features of the episode is successful, the odor recognition and episodic retrieval collapse into a unique memory process that begins as soon as the participants smell the odors. However, further investigations are needed to validate this observation. The use of cerebral imaging techniques represents the ideal tool to test it. We hypothesize that the neural signature of the successful retrieval of episodic information will be observed from the mere odor perception.

### Conflict of interest statement

The authors declare that the research was conducted in the absence of any commercial or financial relationships that could be construed as a potential conflict of interest.

## Abbreviations

CR: Correct rejection
FA: False alarm
WWW: Retrieval of the three dimensions (*What*, *Where*, *Which context*) of the episode
WWhich: Retrieval of the *What* and *Which context* dimensions of the episode
WWhere: Retrieval of the *What* and *Where* dimensions of the episode
What: Retrieval of the *What* dimension of the episode
